# A comparative study of robot-assisted navigation versus C-arm fluoroscopy in percutaneous pedicle screw fixation for the treatment of thoracolumbar fractures

**DOI:** 10.1038/s41598-025-12549-8

**Published:** 2025-07-26

**Authors:** Lichuan Liang, Chen Zhao, Lei Luo, Liehua Liu, Pei Li, Yongjian Gao, Qiang Zhou

**Affiliations:** Department of spine surgery, The Third Affiliated Hospital of Chongqing Medical University, Chong Qing, China

**Keywords:** Robot-assisted navigation, C-arm fluoroscopy, Thoracolumbar fractures, Percutaneous pedicle screw fixation, Screw placement accuracy, Medical research, Clinical trial design

## Abstract

To evaluate the clinical efficacy of ZhuZheng robot-assisted versus C-arm fluoroscopy-guided percutaneous pedicle screw fixation (PPSF) in the treatment of thoracolumbar burst fractures. A retrospective analysis was conducted on 86 patients with thoracolumbar burst fractures treated at our institution between March 2022 and August 2023. The cohort included 46 males and 40 females, aged 27 to 69 years. Patients were assigned to either the robot-assisted group (*n* = 41) or the conventional C-arm fluoroscopy group (*n* = 45) according to intraoperative navigation method. Baseline characteristics, including gender ratio and body mass index (BMI), were comparable between the two groups (*P* > 0.05). All patients underwent segmental fixation. Intraoperative parameters such as fluoroscopy frequency, operative time, and estimated blood loss were recorded. The accuracy of screw placement was assessed using postoperative CT at day 3 and graded according to the Gertzbein-Robbins scale. Pain was evaluated using the Visual Analogue Scale (VAS) preoperatively and at 1 day, 3 days, and 1 month postoperatively. Radiological assessments included Cobb angle and anterior vertebral height ratio at baseline, 3 days, 1 month, and 6 months postoperatively. Statistical analyses were performed using the t-test and Mann-Whitney U test. The robot-assisted group had significantly shorter operative time, reduced intraoperative blood loss, lower radiation dose, and fewer fluoroscopy exposures compared to the conventional group (all *P* < 0.05). No perioperative complications occurred in either group during follow-up. The screw placement accuracy (grades A + B) was significantly higher in the robot-assisted group (98.4%, 242/246) than in the conventional group (90.4%, 244/270; *P* < 0.05). VAS pain scores at postoperative day 1 and day 3 were significantly lower in the robot-assisted group; no significant difference was observed at 1 month. There were no significant intergroup differences in the postoperative Cobb angle or anterior vertebral height ratio at any time point (*P* > 0.05). Robot-assisted orthopedic surgery demonstrates significant advantages in improving screw placement accuracy, reducing intraoperative blood loss, shortening operative time, and minimizing radiation exposure and fluoroscopy frequency, thereby offering improved clinical outcomes in the management of thoracolumbar burst fractures.

## Introduction

Thoracolumbar fractures are predominantly caused by high-energy trauma and are frequently associated with spinal instability^1^, accounting for over 50% of all spinal fractures^2^. Clinically, affected patients often present with localized pain and restricted mobility; in severe cases, neurological deficits such as motor, sensory, or sphincter dysfunction may occur^3^. Prompt and effective reduction and fixation are critical for restoring spinal stability, preventing further neurological impairment, maintaining spinal alignment, and avoiding the development of kyphotic deformity^4^. Pedicle screw fixation represents the standard of care for thoracolumbar burst fractures^5^. While traditional open posterior approaches provide reliable reduction and strong fixation, they require extensive paraspinal muscle dissection and retraction, which may increase intraoperative blood loss, exacerbate soft tissue trauma, and contribute to postoperative complications such as paraspinal muscle atrophy, chronic pain, and limited spinal function. As a minimally invasive technique, percutaneous pedicle screw fixation (PPSF) offers several advantages, including smaller incisions, reduced soft tissue damage, less intraoperative bleeding, and faster postoperative recovery. This technique utilizes percutaneous insertion of pedicle screws combined with appropriate reduction maneuvers, thereby effectively restoring vertebral body height and normal spinal alignment, while maintaining fixation strength and significantly minimizing surgical trauma^6^. Conventional PPSF techniques primarily rely on intraoperative fluoroscopic guidance for screw placement, which is associated with increased radiation exposure, operator-dependent accuracy, and a steep learning curve^7^. The advent of orthopedic surgical robots has introduced new opportunities for PPSF. Robot-assisted navigation systems enhance the accuracy of screw placement, reduce the number of intraoperative fluoroscopy exposures and overall radiation dose, shorten operative time, and improve procedural safety^8^. However, robot-assisted technology also presents certain limitations, such as high acquisition and maintenance costs, increased technical demands on the surgical team^9^, risk of equipment malfunction^10^, and relatively restricted surgical indications. Additionally, the introduction of robotic systems increases the complexity of operating room setup and may not be feasible in certain emergency situations^11^. At present, conventional fluoroscopy-guided PPSF remains the mainstay in clinical practice due to its technical maturity, relative simplicity, and cost-effectiveness. Although robot-assisted PPSF demonstrates clear advantages in terms of technical precision and safety, the selection of surgical approach must consider patient-specific factors, institutional resources, and economic implications. In this retrospective comparative study, we systematically evaluate the clinical efficacy, safety, and cost-effectiveness of conventional fluoroscopy-guided versus robot-assisted PPSF for the treatment of thoracolumbar burst fractures, with the aim of providing evidence-based guidance for surgical decision-making in clinical practice.

To our knowledge, this is the first prospective comparative study evaluating the ZhuZheng robotic platform (Jiangsu ZhuZheng Robot ) in a retrospective analysis of adult thoracolumbar burst-fracture patients.The ZhuZheng system received Class III market authorization from China’s National Medical Products Administration (NMPA; Registration No.20223010268) on 25 February 2022, permitting clinical use in humans in mainland China.

## Materials and methods

### General information

This retrospective cohort study included patients with thoracolumbar burst fractures who underwent surgical treatment at the Third Affiliated Hospital of Chongqing Medical University between March 2022 and August 2023. Written informed consent was obtained from all participants, and the study protocol was approved by the institutional ethics committee.

Inclusion criteria were as follows: (1) single-segment thoracolumbar fracture (T10–L2) within 14 days of injury; (2) Thoracolumbar Injury Classification and Severity Score (TLICS) ≥ 4; (3) bone mineral density T-score ≥-2.5 (normal or osteopenia); (4) intact pedicle anatomy without significant anomalies; (5) AO Spine fracture classification type A (compression fracture) or type B1 (flexion-distraction injury with anterior longitudinal ligament disruption)^12^; (6) clear clinical presentation of thoracic or back pain without neurological deficits.

Exclusion criteria included: (1) osteoporotic compression fractures or pathological fractures secondary to tumor or infection; (2) severe cardiopulmonary dysfunction or other contraindications to surgery; (3) pedicle destruction or anatomical variation precluding pedicle screw fixation; (4) neurological deficits requiring spinal canal decompression; (5) congenital spinal canal stenosis or multilevel spinal fractures.

Patients were allocated into two groups based on the surgical technique: the experimental group (robot-assisted percutaneous pedicle screw fixation, *n* = 41) and the control group (C-arm fluoroscopy-guided percutaneous pedicle screw fixation, *n* = 45). Patient ages ranged from 27 to 69 years. Baseline characteristics for both groups are presented in Table 1.

### Surgical procedures

All procedures were performed by the same surgical team, and intraoperative fluoroscopic guidance was provided by a single radiology technician to ensure procedural consistency.

For the experimental group, the Chinese ZhuZheng robotic system was used to guide percutaneous pedicle screw placement^13,14^. Preoperative CT parameters included a slice thickness ≤ 1.5 mm, no interslice gap, 1:1 interval, spiral scanning mode, image matrix of 512 × 512 pixels, and DICOM format for data transfer. Device calibration was performed prior to surgery to verify normal operation of both the C-arm and robotic systems, validate the data transmission (wired or wireless), and calibrate the laser localization system. All equipment was covered with sterile drapes and arranged contralaterally to the surgeon; the C-arm and robot were spaced approximately 10 cm apart, with a 5 cm gap from the operating table.

**Fig. 1:**
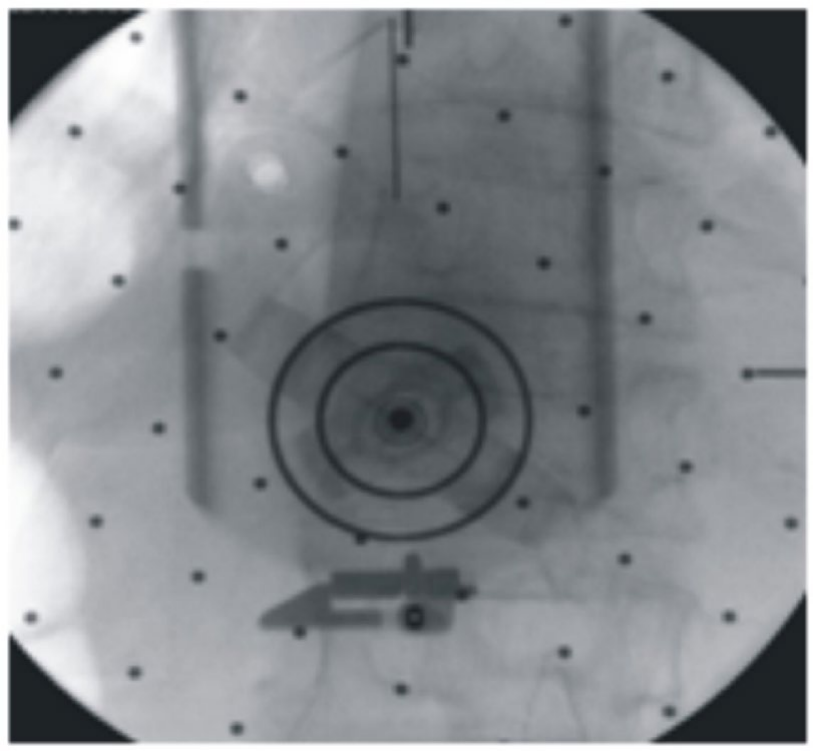
Intraoperative localization: axial fluoroscopy confirms that the projected entry point falls within the innermost circular marker. Entry points that do not exceed the boundary of the inner ring indicate precise puncture localization.

Surgical steps were as follows:

1. Preoperative anteroposterior and lateral fluoroscopy was used to identify the anatomical location of the fractured vertebra;
2. The percutaneous entry point was determined using three-dimensional preoperative planning;
3. The robot was positioned with the C-arm in axial alignment and the trajectory locator installed for initial localization;
4. Under fluoroscopy, the double ring markers were identified, and the robot was precisely aligned until the rings overlapped (rotational error ≤ 0.25°);
5. A small skin incision was made at the predetermined entry point, and the puncture needle was inserted under robotic guidance to the bony surface;
6. The needle was gently tapped into place and its entry point confirmed under axial fluoroscopy within the innermost ring(Figure 1);
7. Pedicle screws were inserted;
8. Fracture reduction was achieved by connecting the screws to the reduction instruments.

For the control group, C-arm fluoroscopy-guided percutaneous pedicle screw fixation was performed. Patients were placed in the prone position under general anesthesia. The target vertebra and adjacent segments were localized by fluoroscopy. Soft tissue channels were sequentially dilated, and pedicle screws were accurately inserted under fluoroscopic guidance. Fracture reduction was then achieved by connecting the screws to the reduction instruments.

To enhance fixation stability, all patients underwent pedicle screw insertion at the fractured vertebra (non-fusion technique, with scheduled hardware removal after healing). For patients with significant vertebral compression, intraoperative positional reduction under general anesthesia was performed to help restore vertebral height.

### Outcome measures

Screw placement accuracy was evaluated by CT scan at 3 days postoperatively using the Gertzbein-Robbins classification: grade A, screw entirely within the pedicle; grade B, cortical breach < 2 mm; grade C, breach 2–4 mm; grade D, breach 4–6 mm; grade E, breach > 6 mm. Grades A and B were considered accurate placement.

Operative parameters included operative time, intraoperative blood loss, number of fluoroscopic exposures, perioperative complications (such as nerve root injury, dural tear, wound infection, hematoma formation), and postoperative hospital stay.

Pain assessment was performed using the Visual Analogue Scale (VAS) preoperatively, and at 1 day, 3 days, and 1 month postoperatively for thoracic and lumbar back pain.

Radiographic evaluation included measurement of the kyphotic Cobb angle and anterior vertebral height ratio preoperatively, and at 3 days, 1 month, and 6 months postoperatively. The Cobb angle was defined as the angle between the upper endplate of the vertebra above and the lower endplate of the vertebra below the fractured segment. The anterior vertebral height ratio was calculated as:

$$\left( {\begin{array}{*{20}c} {anterior\;height\;of} \\ {fractured\;vertebra} \\ \end{array} } \right)\;/\frac{{\left( {\begin{array}{*{20}c} {anterior\;height\;of\;} \\ {upper\;adjacent\;vertebra} \\ \end{array} + \begin{array}{*{20}c} {anterior\;height\;of} \\ {lower\;adjacent\;vertebra} \\ \end{array} } \right)}}{2} \times 100\%$$

Recovery of vertebral height was defined as the postoperative anterior height ratio minus the preoperative ratio.

### Statistical analysis

Statistical analyses were performed using SPSS version 25.0 (IBM Corp., Armonk, NY, USA). Continuous variables are presented as mean ± standard deviation (SD). Normality was assessed using the Shapiro-Wilk test. For normally distributed data, intergroup comparisons were conducted with independent-samples t-tests, and intragroup comparisons with paired t-tests; for non-normally distributed data, the Mann-Whitney U test was used. Categorical variables were analyzed using the χ² test or Fisher’s exact test as appropriate. A P-value < 0.05 was considered statistically significant.

## Results

### Baseline characteristics and perioperative outcomes

A total of 41 patients were included in the robot-assisted group and 45 in the conventional group. All patients were followed for at least 12 months. There were no significant differences between the two groups in terms of gender distribution, age, body mass index (BMI), or distribution of surgical segments (*P* > 0.05; Tables 1 and 2).

Perioperative outcomes demonstrated that the robot-assisted group had significantly shorter operative time, reduced intraoperative blood loss, fewer fluoroscopic exposures, and lower radiation dose compared to the conventional group (Table 1). The difference in postoperative length of hospital stay between the groups was not statistically significant (*P* = 0.115).

No intraoperative complications such as nerve root injury or dural tears occurred in either group, and no cases required conversion to open surgery. During follow-up, there were no incidences of wound infection, implant loosening, or device breakage. All patients underwent successful removal of internal fixation devices 6 to 12 months after surgery.

**Table 1:** Comparison of baseline characteristics and perioperative parameters between the two Groups.

Group	Number of patients	Gender (M/F)		Age (years)		Operation time (minutes)	Blood loss (ml)	Fluoroscopy times	Radiation dose(µGy·m²)	Postoperative hospital stay (days)	Postoperative hospital stay (days)	BMI
Experimental group	41	22/19		51.87 ± 9.24		89.35 ± 9.10	66.25 ± 17.50	9.24 ± 2.57	165.53 ± 40.62	5.42 ± 1.38	23.7 ± 2.0	23.7 ± 2.0
Control group	45	24/21		51.12 ± 12.85		116.78 ± 15.95	138.20 ± 26.80	18.48 ± 3.73	348.83 ± 87.34	7.25 ± 1.28	22.9 ± 1.8	22.9 ± 1.8
P-value		0.842		0.752		0.003	0.001	0.002	0.002	0.115	0.400	0.400

**Table 2:** Distribution of surgical segments between the two Groups.

Group	T10	T11	T12	L1	L2	Distribution of Surgical Segments
Experimental group	2	4	25	7	3	
Control group	3	5	24	9	4	
Group						0.834

### Accuracy of pedicle screw placement

A total of 246 screws were inserted in the robot-assisted group and 270 screws in the conventional group. According to the Gertzbein-Robbins classification, the accuracy rate (grades A + B) was significantly higher in the robot-assisted group (98.4%, 242/246) compared to the conventional group (90.4%, 244/270; *P* = 0.003; Table 3).

**Table 3:** Comparison of pedicle screw placement accuracy between the two Groups.

Group	Number of screws	GradeA	GradeB	GradeC	GradeD	Excellent screw placement rate
Experimental group	246	240	2	4	0	98.4%
Control group	270	228	16	25	1	90.4%
P-value						0.003

### Radiological assessment

There were no statistically significant differences between the two groups in terms of anterior vertebral height ratio and kyphotic Cobb angle at any time point, either preoperatively or postoperatively (*P* > 0.05). Both groups exhibited significant improvements in anterior vertebral height ratio and correction of kyphotic Cobb angle after surgery, which remained stable during follow-up (Table 4).

**Table 4:** Radiological data of the two Groups.

Group	Numbers	Relative height of vertebral anterior edge (%)				Kyphosis Cobb angle (°)			
		Preoperative	3 days postoperative	1 month postoperative	6 months postoperative	Preoperative	3 days postoperative	1 month postoperative	6 months postoperative
Experimental group	41	63.9 ± 5.6	90.4 ± 1.3	89.7 ± 1.4	88.9 ± 1.6	18.4 ± 3.0	6.1 ± 1.5	6.3 ± 1.4	6.8 ± 1.3
Control group	45	64.2 ± 13.1	90.0 ± 1.7	89.5 ± 2.1	88.9 ± 1.8	18.6 ± 2.6	6.1 ± 1.2	6.3 ± 1.1	6.9 ± 1.3
P-value		0.11	0.72	0.16	0.57	0.43	0.21	0.48	0.88

### Comparison of clinical efficacy between the two groups

There was no difference in VAS scores between the two groups before surgery and one month after surgery (*P* > 0.05). However, there were statistically significant differences at one day and three days after surgery (*P* < 0.05) (Table 5).

Table 5—Pain Scores of the Two Groups.

**Table 5:** Thoracic and back pain scores of the two Groups.

Group	Numbers	Pain score (VAS)			
		Preoperative	1 days postoperative	3 days postoperative	1 months postoperative
Experimental group	41	6.55 ± 1.05	2.58 ± 0.50	1.10 ± 0.60	0.70 ± 0.40
Control group	45	6.68 ± 0.90	3.90 ± 0.65	1.85 ± 0.55	1.05 ± 0.50
P-value		0.880	0.000	0.008	0.065

 (Fig. 2).

**Fig. 2:**
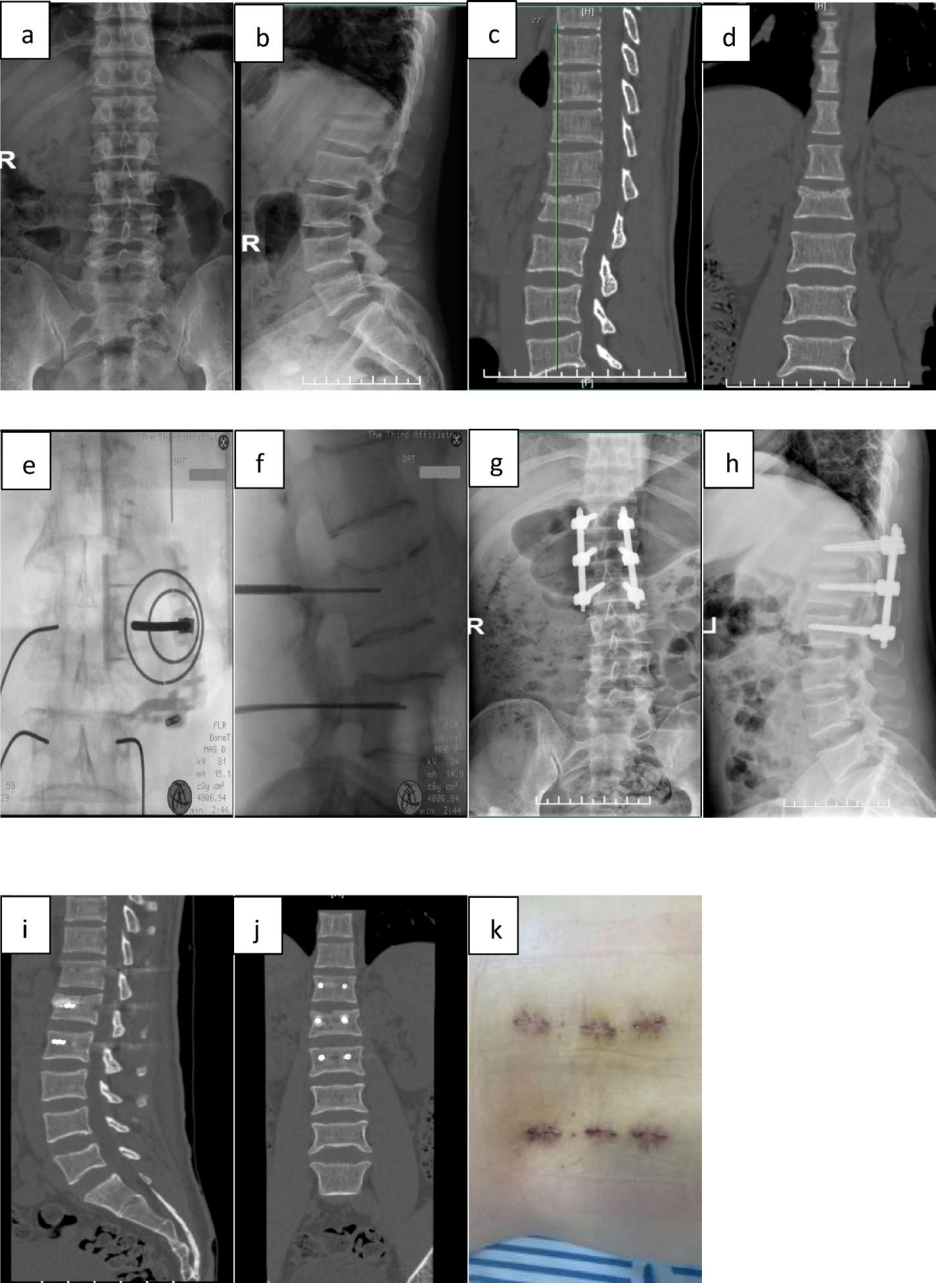
Representative case from the robot-assisted group, a 34-year-old male patient with an L1 burst fracture resulting from a fall. a, b: Preoperative anteroposterior and lateral X-rays showed a burst fracture of the L1 vertebral body. c, d: Preoperative CT revealed fracture of the anterior column and the upper endplate of L1. e, f Intraoperative images demonstrate robot-assisted navigation and screw trajectory planning. f, h: Follow-up radiographs at 3 days postoperatively showed satisfactory fracture reduction. i, j: Postoperative CT at 3 days confirmed that all six screws were grade A. k: Wound condition at 3 days postoperatively during dressing change.

## Discussion

### Advantages of minimally invasive percutaneous surgery

Although advances in surgical techniques and increased surgeon experience have significantly reduced the incidence of complications such as paraspinal muscle ischemia and chronic low back pain following open procedures, minimally invasive percutaneous spinal surgery further minimizes incision size and soft tissue disruption through specialized instrumentation and techniques. Compared with traditional open approaches, this method avoids extensive paraspinal muscle stripping and trauma, resulting in a markedly lower incidence of postoperative complications. Percutaneous pedicle screw fixation (PPSF) has seen increasing application in the treatment of thoracolumbar fractures without neurological injury.

However, conventional minimally invasive procedures are limited by the restricted surgical field, which precludes precise anatomical orientation based solely on visual landmarks^15^. Therefore, intraoperative fluoroscopic guidance is indispensable for accurate screw placement during PPSF. Repeated fluoroscopic imaging and incremental adjustment of entry points are often required to achieve optimal placement, subjecting both patients and medical personnel to increased radiation exposure^16^. Moreover, breaches in the pedicle cortex during screw insertion can result in severe complications^17^, thus restricting the widespread adoption of minimally invasive spinal surgery. The introduction of robotic-assisted technology has substantially addressed these limitations. Our study demonstrated that the number of intraoperative fluoroscopy exposures in the robot-assisted group was significantly reduced (9.24 ± 2.57 vs. 18.48 ± 3.73 in the control group, *P* < 0.05), leading to decreased radiation exposure and improved operative efficiency. Robotic systems enable surgeons to achieve higher precision even in complex anatomical scenarios. The screw placement accuracy in the robot-assisted group reached 98.4%, significantly surpassing the 90.4% accuracy observed in the conventional group (*P* < 0.05), confirming that robotic assistance enhances screw placement precision and lowers the risk of postoperative complications^18^. These improvements contribute to increased safety and effectiveness in surgical outcomes. Nevertheless, robot-assisted PPSF does require a certain learning curve for optimal proficiency^19^.Previous studies have shown that with standardized training, surgeons typically reach a plateau in operative time and blood loss after approximately 20 cases^19^. However, the duration of the learning curve varies depending on the surgeon’s prior experience and the complexity of cases^20^. Multiple navigation systems are currently available for orthopedic procedures, including CT-based navigation, optical navigation, robotic navigation, electromagnetic navigation, and augmented reality-based systems, each with its own advantages, limitations, and specific indications^21^. Collectively, our findings and current evidence indicate that robot-assisted PPSF confers significant clinical benefits in the management of thoracolumbar fractures by enhancing surgical accuracy, reducing intraoperative blood loss, and shortening operative time. This technique thus provides a safer and more effective therapeutic option for patients.These findings expand the evidence base by providing head-to-head outcome data for ZhuZheng—data that were previously unavailable, and support its scalability beyond early feasibility reports.

### Clinical and biomechanical advantages of pedicle screw placement at the fractured vertebra

All patients in this study underwent non-fusion percutaneous pedicle screw fixation, with screws placed at the level of the fractured vertebra, and subsequent removal of the fixation device after healing. The clinical efficacy of screw placement at the fractured vertebra is primarily reflected in the restoration of vertebral body height and the reconstruction of spinal stability^22^.

Screw insertion at the fractured vertebra involves placement in the anteroinferior region of the vertebral body under fluoroscopic guidance. By adjusting the angle between the screws and connecting rods, tightening the screw caps generates an upward lever effect, thereby minimizing mechanical disruption to endplate fragments^23^. Previous studies have reported that the anterior vertebral height can be significantly improved from 69.7% after conventional reduction to 85.1% with this technique^24^. In our study, both groups achieved an anterior vertebral height restoration rate exceeding 90% at 3 days postoperatively, although some loss of height was observed during follow-up. There were no significant differences in the restoration of Cobb angle or anterior vertebral height ratio between the two groups (*P* > 0.05), which may be attributable to standardized surgical techniques and postoperative management.

From a biomechanical perspective, screw placement at the fractured vertebra provides additional fixation points and significantly enhances spinal stability^25^. Precise adjustment of the angle between the screws and connecting rods under fluoroscopic guidance effectively reduces the anterior load on the fractured vertebra, protecting it from further injury^26,27^. In this study, the VAS scores for thoracic and back pain as well as incision pain at 1 and 3 days postoperatively were significantly lower in the robot-assisted group (*P* < 0.05), suggesting that this technique better maintains physiological vertebral alignment and reduces postoperative pain^28^.

By increasing the number of fixation segments, optimizing stress distribution, and improving vertebral height restoration, screw placement at the fractured vertebra offers notable clinical and biomechanical benefits in the minimally invasive treatment of thoracolumbar fractures.

### Accuracy and safety of pedicle screw placement

Accurate pedicle screw placement is a critical determinant of success in spinal surgery^29^. Malpositioned screws can lead to neurovascular injury, postoperative instability, and the need for revision surgery^21,30,31^. In this study, the rate of accurate screw placement was significantly higher in the robot-assisted group (98.4%) than in the conventional group (90.4%; *P* < 0.05), highlighting the advantage of robotic technology in enhancing screw placement accuracy.

Conventional C-arm fluoroscopy-guided pedicle screw placement is highly dependent on the surgeon’s experience and is limited by the restricted surgical field, often resulting in suboptimal screw positioning. Previous studies have reported that the accuracy of screw placement using conventional techniques ranges from 69–94%^32^, whereas robot-assisted techniques can achieve accuracy rates of 94.5–99%^33^. Our findings are consistent with this trend. Furthermore, the robot-assisted group required significantly fewer intraoperative fluoroscopic exposures (9.24 ± 2.57 vs. 18.48 ± 3.73, *P* < 0.05), thereby reducing radiation exposure for both patients and medical staff. Robotic navigation offers precise, reproducible positioning, minimizes surgical error, and lowers the incidence of postoperative complications^34^. Additionally, the literature indicates that robotic guidance not only improves screw accuracy but also effectively protects the proximal facet joints and reduces the risk of neurological, vascular, and muscular injury^8^. No severe complications occurred in either group in this study, further confirming the safety of robotic assistance in spinal surgery.

In summary, robot-assisted pedicle screw placement offers significant advantages in improving accuracy and ensuring surgical safety^35^. The adoption of this technology not only enhances clinical outcomes but also contributes to the standardization and refinement of spinal surgical procedures. Further research is warranted to explore the utility of robotic assistance in various spinal pathologies and to continue evaluating its long-term clinical effectiveness and safety.

### Research limitations and future directions

This study has limitations and directions for future improvement. Firstly, the sample size of only 86 patients may not be sufficient to generalize the results to a broader population, and the single source of the sample limits diversity. Secondly, the follow-up period of at least 12 months is reasonable for primary outcomes but may be insufficient for assessing long-term results, such as adjacent segment degeneration or implant failure. Additionally, surgical technique variations, cost-effectiveness analysis, and patient-reported outcomes need further investigation. In the future, larger-scale, multicenter studies should be conducted to evaluate long-term results with extended follow-up periods, comprehensively analyze cost-effectiveness, and include subjective indicators such as patient satisfaction. Furthermore, with technological advancements, the impact of new-generation robotic systems on surgical outcomes should be assessed, along with comparisons with other minimally invasive techniques.

## Data Availability

Data Availability StatementThe data supporting the findings of our manuscript, titled “Comparative Study of Robot-assisted Navigation and C-arm Fluoroscopy in Percutaneous Pedicle Screw Fixation for Thoracolumbar Fractures,” are available upon reasonable request from the corresponding author, Dr. Qiang Zhou, at the Department of Spine Surgery, the Third Affiliated Hospital of Chongqing Medical University, China. The data will be shared with researchers who provide a methodologically sound proposal for replicating the study findings or conducting new, valid research related to the subject matter.To access the data, researchers must follow these steps: Submit a Research Proposal: The research proposal should outline the objectives, methods, and expected outcomes of the intended research. This proposal will be reviewed by an independent data access committee to ensure its scientific merit and alignment with the goals of the original study.Review by Data Access Committee: The data access committee will evaluate the research proposal and determine whether it meets the criteria for data sharing. The committee’s decision will be communicated to the researcher within a reasonable timeframe.Secure Data Access: Upon approval, the data will be made available in a secure environment that ensures the protection of sensitive patient information. Researchers will be required to sign a data use agreement that outlines the terms and conditions of data access and use.Acknowledge Source and Provide Citations: Any publications or presentations resulting from the use of the data must acknowledge the original source and provide appropriate citations to our manuscript.Please note that the data are the property of the Department of Spine Surgery, the Third Affiliated Hospital of Chongqing Medical University, China. Any use of the data is subject to the terms and conditions set forth by the institution.For any inquiries or to request access to the data, please contact the corresponding author at 651500@hospital.cqmu.edu.cn. We look forward to facilitating legitimate research endeavors that build upon our work.Sincerely,
